# Alternative Splicing of the N-Terminal Cytosolic and Transmembrane Domains of P2X7 Controls Gating of the Ion Channel by ADP-Ribosylation

**DOI:** 10.1371/journal.pone.0041269

**Published:** 2012-07-27

**Authors:** Nicole Schwarz, Laurent Drouot, Annette Nicke, Ralf Fliegert, Olivier Boyer, Andreas H. Guse, Friedrich Haag, Sahil Adriouch, Friedrich Koch-Nolte

**Affiliations:** 1 Institute of Immunology, University Medical Center Hamburg-Eppendorf, Hamburg, Germany; 2 Inserm, U905, University of Rouen, Institute for Research and Innovation in Biomedicine (IRIB), Normandy, France; 3 Max Planck Institute for Experimental Medicine, Göttingen, Germany; 4 The Calcium Signaling Group, Department of Biochemistry and Signal Transduction, University Medical Center Hamburg-Eppendorf, Hamburg, Germany; Virginia Commonwealth University, United States of America

## Abstract

P2X7 is a homotrimeric ion channel with two transmembrane domains and a large extracellular ATP-binding domain. It plays a key role in the response of immune cells to danger signals released from cells at sites of inflammation. Gating of murine P2X7 can be induced by the soluble ligand ATP, as well as by NAD^+^-dependent ADP-ribosylation of arginine 125, a posttranslational protein modification catalyzed by the toxin-related ecto-enzymes ART2.1 and ART2.2. R125 is located at the edge of the ligand-binding crevice. Recently, an alternative splice variant of P2X7, designated P2X7(k), was discovered that differs from the previously described variant P2X7(a) in the N-terminal 42 amino acid residues composing the first cytosolic domain and most of the Tm1 domain. Here we compare the two splice variants of murine P2X7 with respect to their sensitivities to gating by ADP-ribosylation in transfected HEK cells. Our results show that the P2X7(k) variant is sensitive to activation by ADP-ribosylation whereas the P2X7(a) variant is insensitive, despite higher cell surface expression levels. Interestingly, a single point mutation (R276K) renders the P2X7(a) variant sensitive to activation by ADP-ribosylation. Residue 276 is located at the interface of neighboring subunits approximately halfway between the ADP-ribosylation site and the transmembrane domains. Moreover, we show that naive and regulatory T cells preferentially express the more sensitive P2X7(k) variant, while macrophages preferentially express the P2X7(a) variant. Our results indicate that differential splicing of alternative exons encoding the N-terminal cytosolic and transmembrane domains of P2X7 control the sensitivity of different immune cells to extracellular NAD^+^ and ATP.

## Introduction

Following their release from damaged cells, ATP and NAD^+^ function as danger signals that alert cells of the immune system and guide them to sites of tissue damage [1], [2]. In the extracellular compartment these nucleotides act as ligands for receptors and substrates for ecto-enzymes [3], [4]. P2X7 is a homotrimeric ion channel that can be activated by both, ATP and NAD^+^ [5], [6], [7]. While ATP acts as a soluble ligand, activation of P2X7 by NAD^+^ is mediated by the toxin-related ADP-ribosyltransferases ART2.1 and ART2.2. These ecto-enzymes catalyze the transfer of an ADP-ribose moiety from NAD^+^ to arginine 125 (R125) near the ATP-binding site of P2X7, while releasing nicotinamide [8], [9]. ART2.1 and ART2.2 are encoded by tandem genes on mouse chromosome 7. ART2.1 is expressed predominantly by macrophages and displays high enzyme activity only in the presence of extracellular thiols [10], [11]. ART2.2 is expressed predominantly by T cells and appears to be constitutively active [12], [13].

**Figure 1:**
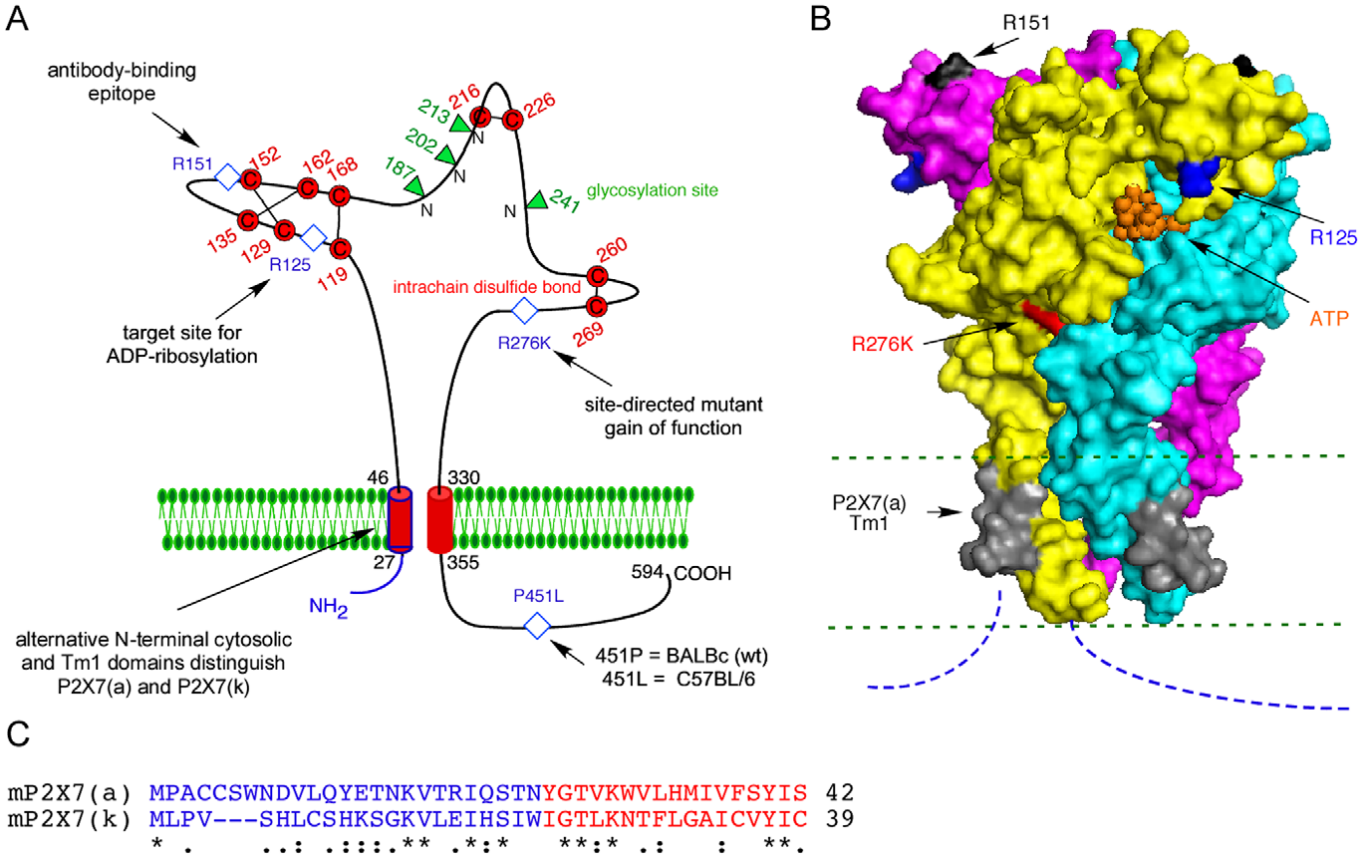
Schematic diagram of P2X7 splice variant and mutants analyzed in this study. The P2X7 (a) and (k) splice variants differ in the N-terminal 42 (a) and 39 (k) amino acids composing the cytosolic N terminal domain and most of the first transmembrane domain (Tm1) [23]. A) The connectivity of cysteine residues (in red) corresponds to that observed in the crystal structure of zebrafish P2X4 [27], [28]. The localization of three arginine (R) residues in the extracellular domain of P2X7 is indicated by blue diamonds (R125 - the site of ADP-ribosylation, R151 - part of the epitope of mAb Hano44, and R276 - conferring a gain of function when mutated to lysine or alanine [8]). The localization of a natural allelic polymorphism in the cytosolic domain that distinguishes C57BL/6 mice from BALB/c and wild-type mice is indicated by a blue diamond [18]. Potential glycosylation sites are indicated by green triangles. B) Model of the crystal structure of P2X7(a) based on the 3D structure of zebra fish P2X4 with bound ATP (pdb code 4dw1) [28]. ATP is indicated in orange, the three subunits of the homotrimer are colored in yellow, cyan, and magenta. The ADP-ribosylation site (R125) is in blue. The binding site of mAb Hano44 (R151) is in black. The gain of function mutant (R276) is in red. Green and blue dashed lines indicate the plane of the membrane and the non-rendered cytosolic domains, respectively. The last 11 of the 42 N-terminal amino acid residues that distinguish splice variant (a) from P2X7(k) are in grey. The structure was modeled with the corresponding residues of P2X7(a). C) Amino acid sequence alignment of the N-terminal cytosolic (blue) and Tm1 (red) domains of P2X7(a) and P2X7(k).

Naive T cells and in particular CD4^+^CD25^+^Foxp3^+^ regulatory T cells are highly sensitive to gating of P2X7 by ADP-ribosylation even at low micromolar concentrations of extracelluar NAD^+^ [14], [15]. This allows influx of Ca^2+^ and efflux of K^+^, and induces a cascade of prominent downstream reactions, including the rapid externalization of phosphatidylserine, ADAM-metalloprotease mediated shedding of L-selectin/CD62L, formation of a membrane pore permeable to large molecules (<900 Da) including DNA-staining dyes, and ultimately results in T cell death [16], [17]. A commonly used strain of mice, C57BL/6, carries an allelic variant of P2X7 that encodes a single point mutation (P451L) located in the long cytosolic domain of P2X7. This mutation impedes some of the downstream effector functions induced by gating of P2X7 [18], [19], [20]. C57BL/6 mice also carry a rare allelic variant of the ART2.1 gene, harboring a premature stop codon that prevents expression [21].

While analyzing nucleotide-induced activation of P2X7 in transfected HEK cells, we have previously made some intriguing observations [8]. In HEK cells co-transfected with cDNA constructs for P2X7 and ART2.2 or ART2.1, all of these effects could be induced by, albeit very high, concentrations of ATP, but none of them could be induced by NAD^+^ [8], [22]. Moreover, when analyzing the effect of a range of arginine to lysine mutations, that were originally generated in the extracellular domain of P2X7 to identify the target sites for ADP-ribosylation, we serendipitously discovered that three of these single mutants (R206K, R276K and R277K) could be activated by NAD^+^-dependent ADP-ribosylation [8], [9]. These same mutants also showed a much lower threshold for activation by ATP. The properties of these "gain of function" mutations were reminiscent of the P2X7 responses observed in murine T cells. In contrast, P2X7 on murine macrophages, akin to P2X7 on HEK cells, could be gated only by high concentrations of ATP but not by ADP-ribosylation [22].

Recently, an alternative splice variant of P2X7 was discovered in rat and mouse [23]. This splice variant, designated P2X7(k), differs from the previously described variant P2X7(a) in the N-terminal 42 amino acid residues composing the N-terminal cytosolic domain and most of the Tm1 domain (**Fig. 1**). The rat P2X7(k) variant was shown to be more sensitive to the P2X7 agonist Bz-ATP, have a slower deactivation time upon removal of agonist, and to have a higher propensity to form pores [23]. Here, we compared the two murine splice variants with respect to sensitivity to gating by ADP-ribosylation in transfected HEK cells. Our results demonstrate that P2X7(k) but not P2X7(a) is sensitive to gating by ADP-ribosylation. Moreover, we show that naive and regulatory T cells preferentially express the more sensitive P2X7(k) variant, whereas macrophages express the less sensitive P2X7(a) variant. Our results indicate that differential splicing of alternative exons encoding the N-terminal cytosolic and transmembrane domains of P2X7 dictate the sensitivity of different immune cells to extracellular NAD^+^ and ATP.

**Figure 2:**
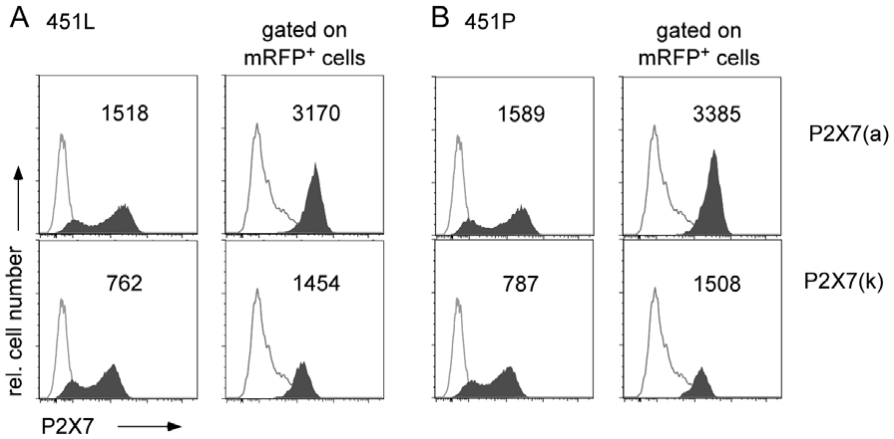
The P2X7(a) splice variant shows two-fold higher cell surface expression levels in transfected HEK cells than the P2X7(k) variant. HEK cells were co-transfected with expression constructs for mRFP and P2X7(a) (upper panels) or P2X7(k) lower panels. A) P2X7 splice variants carrying the 451L allele found in C57BL/6 mice; B) P2X7 splice variants carrying the 451P allele found in BALBc mice. FACS-analyses of native P2X7 on the surface of HEK cells were performed 20 h after transfection using Alexa647-conjugated monoclonal antibody Hano44 (shaded histograms) or an isotype control (open histograms) [26]. Gating was performed on all cells (left panels) or on mRFP-positive cells. Numbers indicate mean fluorescent intensities of P2X7 staining. Results are representative of four independent experiments.

## Materials and Methods

### Mice

C57BL/6 and BALB/c mice were obtained from Centre d’Elevage Janvier or from The Jackson Laboratory. All animal experiments have been conducted according to relevant national and international guidelines and have been approved by the local institutional ethic committees (Animal Care and Use Committee, Behoerde fuer Soziales, Familie, Gesundheit und Verbraucherschutz - Lebensmittelsicherheit und Veterinaerwesen – animal permit number G08/067_ADP, and Comité régional d’éthique en expérimentation animale de Normandie, authorization number N/03-11-09/22/11-12).

### Preparation and Analysis of Primary Lymphocytes

Single-cell suspensions were prepared from lymph nodes and spleens in cold (4°C) RPMI 1640 medium by gentle dissection and passage through Nitex membrane (125-µm mesh, Tetko). Conventional and regulatory T cells were purified with magnetic beads as described previously [15], [16] using the CD4 negative isolation kit (Invitrogen) and positive selection using anti-CD25-conjugated magnetic beads (Milteny Treg purification kit). Purity of T cell subpopulations was always >95% as verified by FACS analyses.

**Figure 3:**
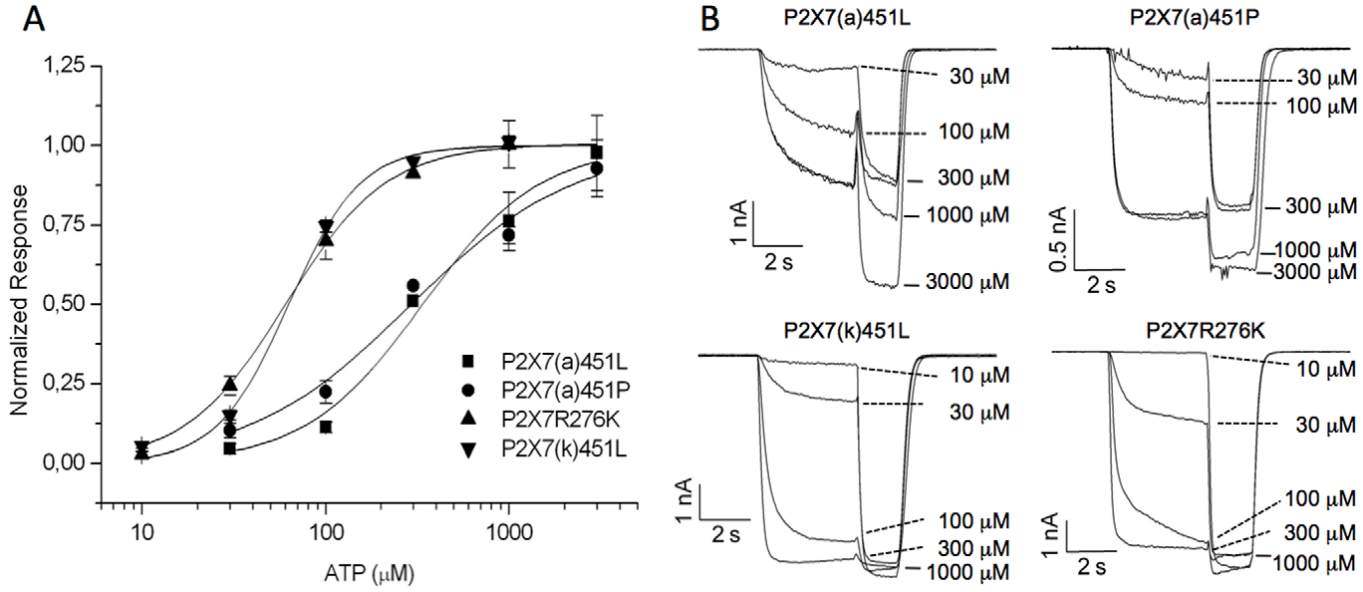
P2X7(k) shows higher sensitivity to gating by ATP than P2X7(a). HEK cells transfected with the indicated P2X7 constructs were analyzed 1–3 days after transfection and clamped at -70 mV [23]. (A) Comparison of dose response curves for ATP. The values of the test concentrations were calculated in reference to the value obtained with a 300 µM reference concentration that was given immediately before or after the test concentration. Dose response curves were fit to the Hill equation and subsequently normalized to their maximal values. The following EC_50_ values and Hill coefficients were obtained: P2X7(k)451L: 63±4 µM, P2X7(a)276K: 61±4 µM, P2X7(a)451P: 338±89 µM, and P2X7(a)451L 292±117 µM (n = 3–8 for each data point). (B) Representative current traces and recording protocol for the dose response analysis. The indicated test concentrations and a 300 µM reference concentration of ATP were consecutively applied to account for run-up/run-down of receptor currents during the experiment. Results are representative of four independent experiments.

### Expression Vectors and Cell Transfections

Expression vectors for ART2.1, ART2.2, P2X7(a)276K, and CD62L were cloned as described previously [18], [22], [23], [24]. The expression construct for monomeric red fluorescent protein (mRFP) was from Clontech Laboratories Inc. (Palo Alto, CA, USA). P2X7(a) and P2X7(k) constructs were subcloned into the pCDNA3.1 vector (Invitrogen, Karlsruhe, Germany). The 451P mutation was introduced by site directed mutagenesis. For patch clamp analysis, P2X7 variants were subcloned into a modified (addition of a second CMV promoter from the pAdTrackCMV vector [25] pTurbo FP635-N vector (Evrogen, Moscow, Russia), to co-express mRFP as a transfection marker. For patch clamp analysis, cells were transfected with 1 µg DNA/well on six-well plate using lipofectamin (Invitrogen). For FACS analyses and live cell imaging, cells were transfected in solution with 2.5 µg DNA for cell surface antigens and 0.5 µg DNA for mRFP per 10^6^ cells using the jetPEI transfection reagent (Q-Biogen, Illkirch, France) before plating onto T25 flasks or cover slips.

**Figure 4:**
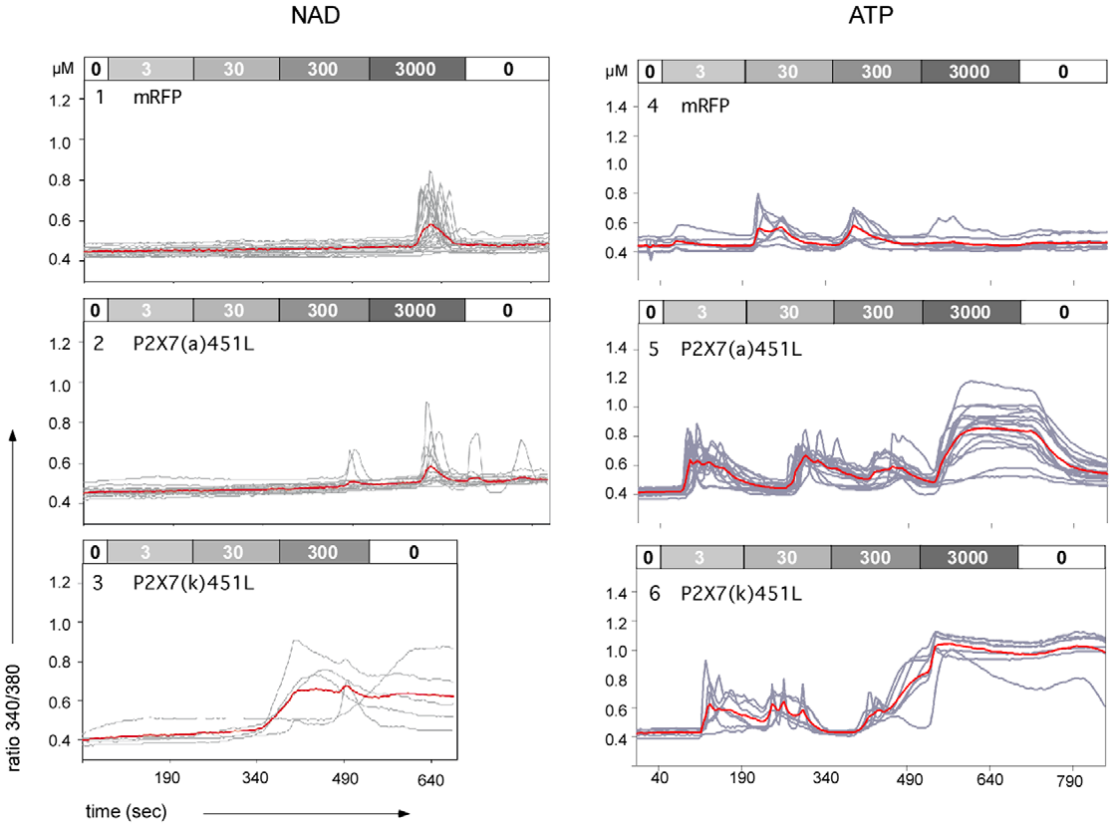
ADP-ribosylation of P2X7(k) induces stable Ca^2+^ signals. HEK cells were co-transfected with expression constructs for mRFP, ART2.2, and the indicated splice variants of P2X7. 20 h post transfection, cells were loaded with the Ca^2+^-sensitive fluorophore Fura-2 before live-cell imaging by fluorescence microscopy at 37°C [8]. Images were captured every 5 sec. At the indicated times, the perfusion buffer was changed to perfuse cells with increasing doses of NAD^+^ (panels 1–3) or ATP (panels 4–6). Ratio images (340/380 nm) were constructed pixel-by-pixel and single cell tracings were obtained using the Openlab software. Grey lines show single cell tracings, red lines the calculated mean. Results are representative of two independent experiments.

### Antibodies and FACS Analyses

The monoclonal anti-P2X7 antibody Hano44, generated by genetic immunization as described previously [26], was conjugated to Alexa488 or Alexa647 (Invitrogen, Carlsbad, CA, USA) according to the manufacturer’s instructions. HEK cells were harvested by trypsinization 20–48 h post-transfection. Cells were washed and then incubated in the absence or presence of the indicated concentrations of NAD^+^ or ATP in DMEM medium containing 1 mM DTT for 60 min at 37°C. Cells were washed and stained with Alexa488, Alexa647 or PE-conjugated antibodies (0.5 µg/2×10^5^ cells/100 µl) and Pacific Orange (PacO, Molecular Probes) for 20 min at 4°C. All measurements were performed on a FACS-Calibur (BD, Franklin Lakes, NJ, USA) and analyzed with the FlowJo software.

**Figure 5:**
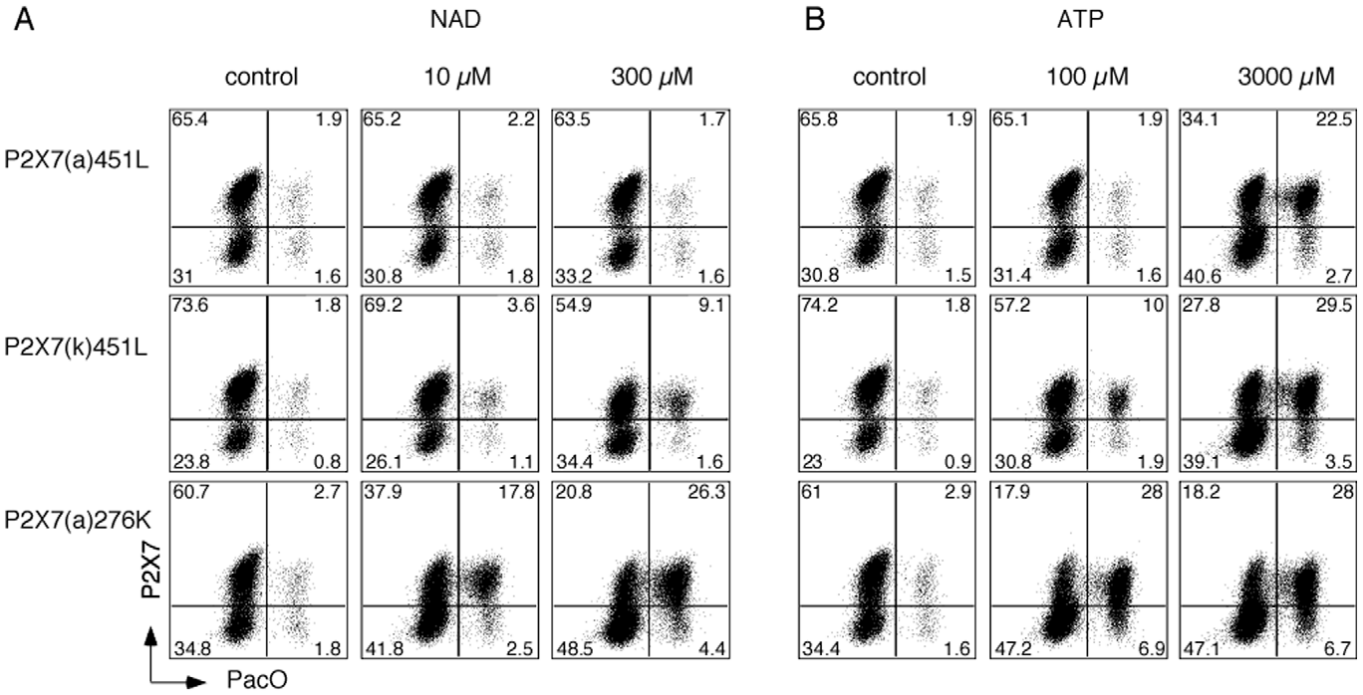
ADP-ribosylation of P2X7(k) induces permeabilization of the cell membrane to the amine-reactive dye PacO. HEK cells were co-transfected with expression constructs for ART2.1 and the indicated splice variants of P2X7, harvested 48 post transfection, and incubated for 30 min in the presence of the indicated concentrations of NAD^+^ (A) or ATP (B) as in Fig. 3. Cells were stained with Alexa647-conjugated anti-P2X7 (Hano44) and amine-reactive Pacific Orange (PacO) and subjected to FACS-analyses. Similar results were obtained with constructs on the BALBc background (451P). Results are representative of two independent experiments.

### Ca^2+^ Imaging by Fluorescence Microscopy

Ratiometric Ca^2+^ imaging was essentially performed as described previously [8], [9]. In short: 20h post co-transfection of HEK cells with expression vectors for P2X7, ART2.2, and mRFP cells were loaded with the ratiometric Ca^2+^ indicator Fura-2 (Calbiochem, EMD Biosciences Inc., San Diego, CA, USA). After loading, cells were subjected to Ca^2+^ imaging using imaging system consisting of a Leica DM-IRBE fluorescence microscope with a 40x objective (1.3 numerical aperture), a monochromator system (polychromator II; TILL Photonics, Graefelfing, Germany) as a light source, and a grayscale CCD camera (type C4742–95-12NRB; Hamamatsu, Enfield, UK). During the experiments, cells were continuously perfused with prewarmed (37°C) buffer (15 mM HEPES, pH 7.4, 140 mM NaCl, 5 mM KCl, 1 mM MgCl_2_, 1.35 mM CaCl_2_, 10 mM glucose, 0.1% BSA) containing the indicated concentrations of ATP or NAD using a perfusion system from Warner Instruments (Hamden, CT, USA). Every 5 s two images were taken at excitation wavelengths of 340 and 380 nm. For calculation of ratio images (340/380 nm) and analysis Openlab software (v3.09; Improvision, Tübingen, Germany) was used.

### Whole Cell Patch Clamping

Whole cell patch clamp recordings from transiently transfected cells were performed at room temperature as previously described [23] using an EPC9 amplifier and Pulse acquisition software (HEKA, Lambrecht, Germany). Patch pipettes (2–3 MΩ) were pulled from Corning 0010 glass (WPI, Sarasota, FL). The pipette solution contained 147 mM NaCl, 10 mM HEPES, and 10 mM EGTA. During the recording, cells were constantly perfused with extracellular solution containing 147 mM NaCl, 2 mM KCl, 2 mM CaCl_2_, 1 mM MgCl_2_, 10 mM HEPES, and 13 mM glucose. Both solutions were adjusted with NaOH to pH 7.3–7.35 and 300–310 mosmol/kg. The membrane potential was clamped at -70 mV. Agonist-containing solutions were automatically applied with slight pressure via an automated valve system (Nanion, Munich, Germany) and a manifold (ALA Scientific Instruments, Farmingdale, NY) positioned above the cell. To account for the observed sensitization and run down of P2X7 receptor currents, data were collected after obtaining stable responses to 300 µM ATP (Fluka); this concentration was also applied as a reference concentration immediately after application of each test concentration. EC_50_ values were calculated from a non-linear fit of the Hill equation to the data using Origin 7.5 software (Origin- Lab Corp., Northampton, MA). Data points are presented as mean ± S.E. from 3–8 cells.

**Figure 6:**
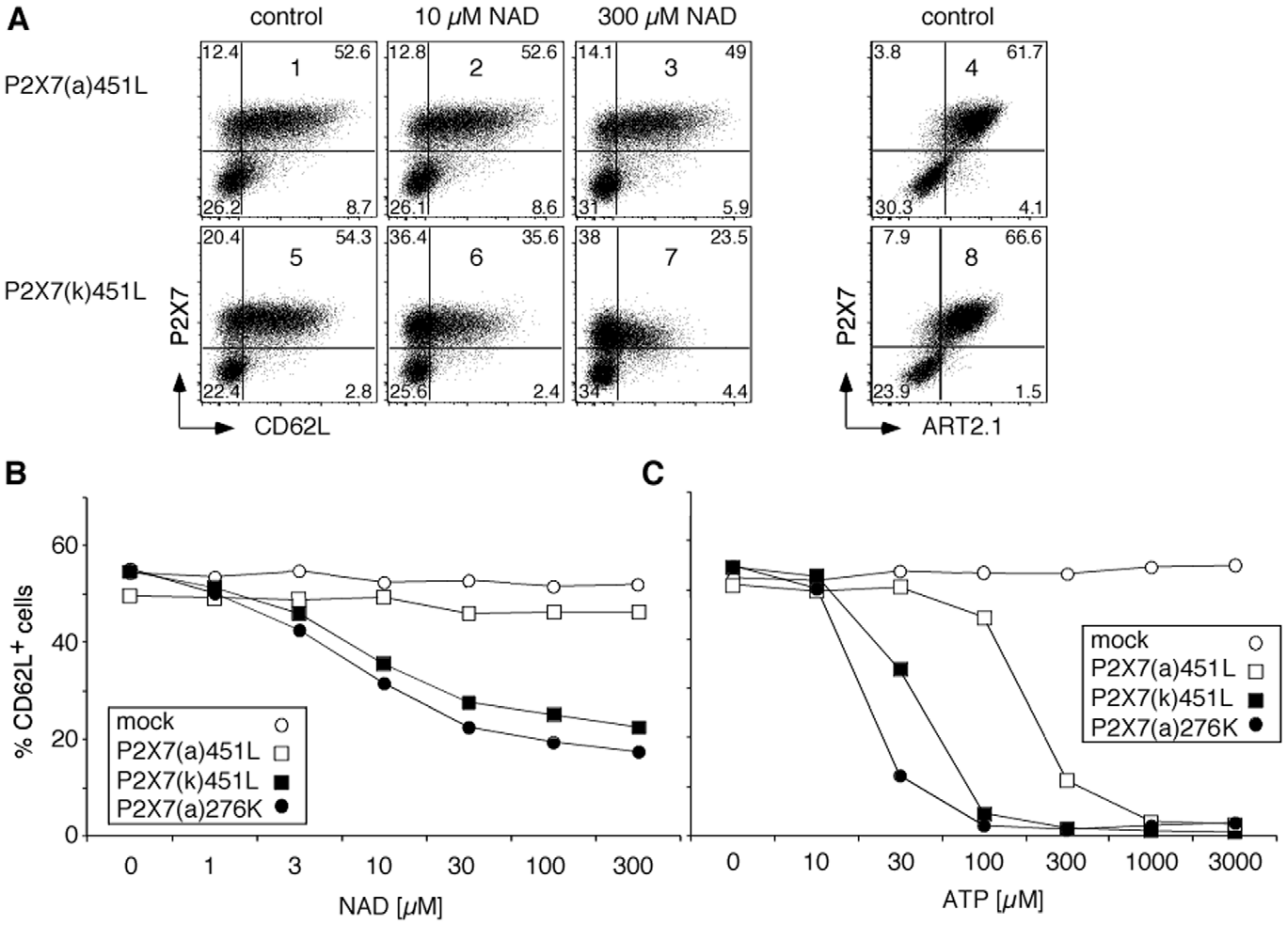
ADP-ribosylation of P2X7(k) induces shedding of CD62L. HEK cells were co-transfected with expression constructs for ART2.1, CD62L, and the indicated splice variants of P2X7 [8]. Cells were harvested 48h after transfection by gentle trypsinization, washed and incubated for 30 min at 37°C in the presence of the indicated concentrations of NAD^+^ or ATP before staining with Alexa647-conjugated anti-P2X7 (Hano44), Alexa488-conjugated anti-ART2.1 (R18A22), and PE-conjugated anti-CD62L (MEL-14) and FACS-analyses. Similar results were obtained with constructs harboring the 451P allele found in BALBc mice. Results are representative of four independent experiments.

### Quantitative Real Time PCR

Total RNA was prepared from using either TRIzol (Invitrogen) for muscle samples or RNAqueous® Kit (Invitrogen) for purified macrophages and T cells. Equivalent amounts of RNA were reverse transcribed using random hexamers (Superscript first strand synthesis system for RT-PCR, Invitrogen). Reverse transcriptase was omitted in negative controls. PCR on mouse cells was performed with primers specific to exon one of P2X7(a) and P2X7(k), respectively (5′- CAC ATG ATC GTC TTT TCC TAC - 3′) and (5′-GCC CGT GAG CCA CTT ATG C -3′) [23]. Antisense primers targeting the common exon 2 were chosen to match each specific forward primer and to give optimal PCR amplification. The chosen antisense primer for detection of P2X7(a) and P2X7(k) were, respectively, (5′-CCT GCA AAG GGA AGG TGT AG-3′) and (5′-CCC ACC CTC TGT GAC ATT CT-3′). The qPCR reaction mixtures were set-up in glass-capillaries using 2 µl cDNA template and the reaction mixture recommended by the kit supplier (FastStart DNA Master SYBR Green I, Roche). The amplification conditions were 40 cycles of 95°C for 10s, 60°C for 10s, and 72°C for 10s using a real time cycler (lightCycler®, Roche). The relative amount of P2X7 variants mRNA for each sample was then normalized against the eEF2 house keeping gene mRNA amount and quantified in arbitrary units by comparison to serial dilution of reference plasmids containing the cloned PCR products. The identity of the PCR products was confirmed by gel analysis and/or sequencing.

**Figure 7:**
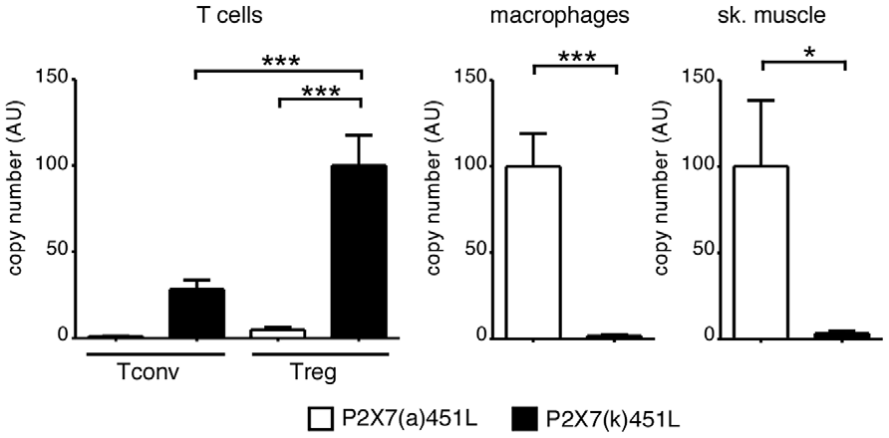
P2X7(k) is the dominant transcript in T cells, P2X7(a) is the dominant transcript in macrophages and muscle cells. Total RNA was prepared from primary splenic and lymph node T cells, peritoneal macrophages, and skeletal muscle tissue from C57BL/6 mice (carrying the 451L allelic variant of P2X7). RNA samples were subjected to quantitative RT-PCR analyses with primers specific to exon one of P2X7(a) or P2X7(k) [23]. Results are representative of four independent experiments for lymphocytes and three independent experiments for macrophages and skeletal muscle. Error bars represent SEM. *, p<0.05; ***, p<0.001.

### Statistical Analysis

All data are shown as mean values and error bars represent SEM. For statistical comparisons, one-way analysis of variance (ANOVA) and Tukey’s post-hoc tests were used. Differences were considered to be statistically significant when p-values were <0.05. All calculations were performed using Prism 5.04 software (Graph-Pad Software, Inc.).

### 3D Modeling

The 3D structure of mouse P2X7 was modeled on the recently published structure of zebrafish P2X4 [27], [28] using Swiss model (URL http://swissmodel.expasy.org).

## Results and Discussion

### In Transfected HEK Cells P2X7(a) Shows Two-fold Higher Cell Surface Expression Levels than P2X7(k)

The availability of a monoclonal antibody recognizing P2X7 in native conformation [26], [29] allows direct quantification of P2X7 exposed on the surface of individual living cells. Western-Blot analyses, in contrast, only provide information about total P2X7 protein levels in a population of cells. In order to assess cell surface expression of the P2X7 splice variants, we transiently transfected HEK cells with cDNA expression constructs and monitored cell surface expression of the P2X7 isoforms by FACS using monoclonal antibody Hano44 that detects a common epitope (encompassing residue R151) on both splice variants (**Fig. 2**) [8], [26]. The efficiency of transfection was monitored by co-transfection with mRFP. While transfection efficiencies were similar for both variants, the mean fluorescence intensities of HEK cells transfected with P2X7(a) (Fig. 2, upper panels) were consistently approximately two-fold higher than those of cells transfected with P2X7(k) (Fig. 2, lower panels), regardless of whether the splice variants carried the 451 allele found in C57BL/6 (451L) (Fig. 2A) or that found in BALBc mice (451P) (Fig. 2B) [18]. The results indicate that assembly and transport to the cell surface of the P2X7(a) variant is more efficient than that of the P2X7(k) variant.

### In Transfected HEK Cells, the P2X7(k) Variant Shows a Five-fold Higher Sensitivity to ATP-Induced Gating than the P2X7(a) Variant

Using whole cell patch clamp recordings of HEK cells transfected with splice variants of rat P2X7, we have previously observed that cells expressing P2X7(k) were 8-fold more sensitive to the agonist Bz-ATP and displayed much slower deactivation kinetics than cells expressing P2X7(a) [23]. In order to assess gating of the mouse P2X7 splice variants, HEK cells transfected with mouse P2X7(a) and P2X7(k) constructs were analyzed 1 to 3 days after transfection and clamped at −70 mV [23] (**Fig. 3**). Upon application of 300 µM ATP, both variants were rapidly activated and reached a constant current within 4 s (Fig. 3B). In contrast to the previously described rat variants, both variants quickly inactivated after removal of the agonist. Dose-response analysis revealed a five-fold higher sensitivity of the P2X7(k) variant for ATP (EC_50_ 60 µM) than the P2X7(a) variant (EC_50_ 300 µM) (Fig. 3A). The previously described R276K gain of function mutant of P2X7(a) [8] also displayed an increased ATP-sensitivity (EC_50_ 60 µM), similar to that of the P2X7(k) variant.

### ADP-ribosylation of the P2X7(k) but not the P2X7(a) Variant Induces Stable Ca^2+^ Signals

We previously noticed that HEK cells co-transfected with P2X7(a) and ART2.2 did not show any Ca^2+^ influx in response to treatment with NAD^+^, but did respond to very high concentrations of extracellular ATP (1 mM) [8], [9]. In striking contrast, the P2X7(a) R276K mutant showed vigorous responses to low concentrations of NAD^+^ [8], [9]. In order to determine whether ADP-ribosylation could gate the P2X7(k) variant, we analyzed free cytosolic Ca^2+^ concentrations in HEK cells loaded with the Ca^2+^ sensitive fluorophore Fura-2 48 h after co-transfection with ART2.2 and P2X7 by live cell imaging (**Fig. 4**). Cells were perfused with increasing concentrations of NAD^+^ or ATP for three-minute intervals, followed by a wash-out of the nucleotide. Consistent with previous observations [8], [22], P2X7(a)/ART2.2 co-transfected HEK cells did not show any detectable responses, even to very high concentrations of NAD^+^ (Fig. 4, panel 2). In striking contrast, HEK cells co-transfected with P2X7(k) and ART2.2, responded with stable Ca^2+^ signals upon perfusion with NAD^+^ concentrations above 30 µM (Fig. 4, panel 3). Similarly, HEK cells transfected with the P2X7(k) variant showed detectable but submaximal responses to 300 µM ATP, while P2X7(a) transfected cells showed responses only at very high levels of ATP (3 mM) (Fig. 4, panels 5, 6). Remarkably, while wash out of ATP was followed by a gradual decrease of Ca^2+^-induced fluorescence in case of P2X7(a)-transfected cells (Fig. 4, panel 5), Ca^2+^-induced fluorescence remained at high levels for more than 3 minutes after wash out of the agonist in case of P2X7(k)-transfected cells (Fig. 4, panel 6), possibly reflecting sustained channel opening or intracellular redistribution of the Ca^2+^ sensor, Fura-2.

### ADP-ribosylation of P2X7(k) Induces Permeabilization of the Cell Membrane to the Amine-reactive Dye Pacific Orange (PacO)

Activation of P2X7 on naive T cells following treatment with extracellular NAD^+^ induces a rapid ART2- and P2X7-dependent externalization of phosphatidylserine, and ultimately, cell death [17], [30]. Amine-reactive dyes are convenient dyes for dead cell discrimination by flow cytometry [31]. These dyes covalently bind to amine groups on proteins. On live cells, only membrane proteins are accessible to these dyes, resulting in dim fluorescence. On cells with damaged membranes, however, these dyes can enter cells and cause an up to fifty-fold increase in fluorescence. In order to assess whether NAD^+^ treatment can induce the uptake of the 750 Dalton amine-reactive dye PacO, we analyzed HEK cells co-transfected with ART2.1 and P2X7 splice variants (**Fig. 5**). A substantial fraction of cells transfected with P2X7(k) responded to NAD^+^ treatment with increased staining by PacO in a concentration-dependent manner (Fig. 5A). In contrast, cells transfected with P2X7(a) did not show a detectable PacO uptake in response to NAD^+^ (Fig. 5A top panels). In this assay, the R276K gain of function mutant showed much stronger responses to both NAD^+^, and ATP than P2X7(k) (Fig. 5 A, B, lower panels).

### ADP-ribosylation of P2X7(k) Induces Shedding of L-selectin (CD62L)

Treatment of naive T cells with extracellular NAD^+^ induces the ART2- and P2X7-dependent shedding of CD62L by an ADAM-like metalloprotease [17], [30]. In order to assess whether the ART2>P2X7> ADAM > CD62L axis could be reconstituted in HEK cells, we co-transfected cells with ART2.1, P2X7 and CD62L (**Fig. 6**). FACS analyses confirmed robust co-expression of ART2.1, P2X7 and CD62L 40 h after transfection (Fig. 6A, panels 1, 4, 7, 8). Remarkably, cells transfected with P2X7(k) responded to NAD^+^ treatment with loss of CD62L from the cell surface in a concentration-dependent manner (IC_50_ 4 µM) (Fig. 6B). Similarly, these cells showed concentration-dependent down-modulation of P2X7 itself (Fig. 6A, panels 4–6). In striking contrast, cells transfected with P2X7(a) did not show any detectable shedding of CD62L in response to NAD^+^ (Fig. 6A panels 1–3). Consistently, HEK cells transfected with the P2X7(k) variant showed a higher sensitivity to ATP-induced shedding of CD62L (IC_50_ 30 µM vs. 180 µM) (Fig. 6C). Again, the sensitivity of the P2X7(k) variant to both, NAD^+^ and ATP, was similar to that of P2X7(a) carrying the R276K gain of function mutation (Fig. 6 B, C).

### P2X7(k) is the Dominant Transcript in Conventional and Regulatory T Cells, P2X7(a) is the Predominant Transcript in Macrophages and Muscle Cells

Given the high sensitivity of P2X7(k) and the insensitivity of P2X7(a) towards NAD^+^ in transfected HEK cells, we wondered whether differential splicing might be used *in vivo* to govern the differential response of different cell types to activation by ATP and NAD^+^-dependent ADP-ribosylation. To address this question we performed quantitative RT-PCR analyses on purified T cell subsets and macrophages using splice variant-specific PCR primers (**Fig. 7**). The results show that T cells contain 20-30-fold higher copy numbers of P2X7(k) *vs.* P2X7(a) transcripts. Remarkably, the reverse holds true for macrophages and muscle cells that contain 30-50-fold higher copy numbers of the less sensitive P2X7(a) transcripts. The higher transcript numbers of P2X7 in regulatory vs. conventional T cell subsets correlates with the previously observed higher sensitivity of Tregs to extracellular ATP and NAD^+^ [14], [15].

### Conclusions

In this study, we compared the sensitivities of P2X7 splice variants differing in the N-terminal cytosolic-Tm1 domain to gating by ATP and NAD^+^-dependent ADP-ribosylation using HEK cells transfected with the ART2 ecto-enzymes and P2X7 splice variants. Our results show that ADP-ribosylation activates the P2X7(k) variant, inducing influx of Ca^2+^, shedding of CD62L, and permeabilization of cells to 750 Da amine-reactive dyes. The P2X7(a) variant, in contrast is insensitive to activation by ADP-ribosylation. The differential sensitivities toward NAD^+^ are mirrored by similar differential sensitivities to activation by ATP. The finding that P2X7(k) responds more vigorously than P2X7(a) (Figs. 3–6) despite lower expression on the cell surface (Fig. 2) suggests that the composition and structure of the N-terminus of P2X7 is more important for gating sensitivity than receptor density on the cell surface. An alternative splice variant lacking the C-terminal tail was recently described for human P2X7 [32]. It will be interesting to determine whether the mouse can generate an isoform lacking the C-terminal tail similar to human P2X7(B) and whether the human can generate an isoform with an alternative N-terminus similar to mouse P2X7(k).

Interestingly, the P2X7(a) variant can be rendered sensitive to gating by ADP-ribosylation by site-directed replacement of a single amino acid residue (R276) at a considerable distance outside of the transmembrane domain [8] (Figs. 5, 6). This residue 276 is located at the interface of neighboring subunits approximately halfway between the ADP-ribosylation site and the transmembrane domains (Fig. 1B). It is tempting to speculate that allosteric modulators may similarly render the P2X7(a) variant sensitive to gating by ADP-ribosylation under (patho)physiological conditions.

We conclude that the composition of the N-terminal cytosolic and first transmembrane domain control gating of P2X7 by ADP-ribosylation in transfected HEK cells. Our results further demonstrate that T cells preferentially express the P2X7(k) variant while macrophages preferentially express the less sensitive P2X7(a) variant. These findings correlate with the previously observed differential sensitivities of these cells to extracellular ATP and NAD^+^ [22]. As the P2X7(k) variant escapes gene deletion in the Glaxo P2X7^−/−^ mice [23], these results also explain why T cells were still found to be sensitive to ATP in these mice while macrophages were rendered insensitive by the same genetic deletion [33]. We conclude that alternative splicing of P2X7 governs the sensitivity of cells to gating of P2X7 by ADP-ribosylation and by ATP.
